# Genetic Diversity and Population Structure of the Native Pulawska and Three Commercial Pig Breeds Based on Microsatellite Markers

**DOI:** 10.3390/genes14020276

**Published:** 2023-01-20

**Authors:** Anna Radko, Anna Koseniuk, Grzegorz Smołucha

**Affiliations:** National Research Institute of Animal Production, Department of Animal Molecular Biology, Krakowska Street 1, 32-083 Balice, Poland

**Keywords:** *Sus scrofa*, STR, genetic differentiation

## Abstract

Swine DNA profiling is highly important for animal identification and parentage verification and also increasingly important for meat traceability. This work aimed to analyze the genetic structure and genetic diversity in selected Polish pig breeds. The study used a set of 14 microsatellite (STR) markers recommended by ISAG for parentage verification in the native Puławska pig (PUL, n = 85) and three commercial pig breeds: Polish Large White (PLW, *n* = 74), Polish Landrace (PL, *n* = 85) and foreign breed Duroc (DUR, *n* = 84). Genetic differentiation among breeds accounted for 18% of the total genetic variability (AMOVA). Bayesian structure analysis (STRUCTURE) indicated that the four distinct genetic clusters obtained corresponded to the four breeds studied. The genetic Reynolds distances (Ɵw) showed a close relationship between PL and PLW breeds and the most distant for DUR and PUL pigs. The genetic differentiation values (*F*_ST_) were lower between PL and PLW and higher between PUL and DUR. The principal coordinate analysis (PCoA) supported the classification of the populations into four clusters.

## 1. Introduction

Maintaining high-quality meat production and the preservation of food safety are directly related to the biodiversity and individual and breed identification of animals. Swine DNA profiling is highly important for animal identification, parentage verification and, more recently, meat traceability [1,2,3]. Pork is the most frequently chosen meat by consumers; therefore, maintaining high-quality standards in pork production is very important—not only for commercial pigs but especially for the population of native pig breeds. Native breed pigs can provide meat that is both high in quality and functional. Local, primitive breeds give rise to native pig breeds. Breed purity was maintained through the careful selection of individuals for mating, while breeding work was based on selective breeding. Many of the traits inherited from primitive ancestors are present in pigs from native breeds, such as adaptability to local environmental conditions, feed availability and extensive farming. In addition to high fertility, the animals have good maternal care and display good breeding performance. The animals live for a long time and are resistant to stress and pathogens. Products obtained from their meat possess unique sensory and nutritional quality. The native breeds of pigs in Poland are White Złotnicka, Złotnicka Spotted and Puławska. Polish native breeds are valued not only for the fact that they are bred in Poland, but also because their meat is used to produce traditional Polish cured meats with specific technological properties and taste qualities. Regarding the chosen Polish native pigs breed to study, the Puławska breed originates from the Lublin region. These pigs have numerous favorable characteristics, such as good health, resistance to disease, low fodder requirements and fattening tendency. Moreover, the Puławska breed is recognized for its early maturation, rapid growth, high feed utilization, high fattening and slaughter values and adaptability to local environmental conditions. Through cross-breeding this pig with the Large White and Berkshire breeds, the Pulawska population was transformed into a dual-purpose fat–meat type. Now, the Puławska breed is used for commercial crossing [4]. Puławska, as a rare breed, was included in the Farm Animals Genetic Resources Conservation Program. Among the Polish local breeds, the distinguishing quality traits of the Puławska pig are emphasized. Meat obtained from these animals is characterized by higher culinary value than that from mass production. Recently, products from this breed have been very popular on the Polish market, e.g., “traditional cold cuts from Puławiak”. In order for authentic Pulawska breed meat and meat products to be verified, it should be included in DNA testing for the individual identification of animals and provision for the retrieval of this information as and when required, which is very important for meat traceability. This means that the meat is produced from an identified animal and has information about its origin. According to ISAG recommendations published in the 1990s, microsatellite markers (short tandem repeats, STR) can be used to prove the parentage of farm animals. Moreover, STRs are applied to study genetic structure and diversity, as well as to track the ancestry of diverse farm animal species [5,6,7,8,9], including pigs [10,11,12,13,14,15,16,17]. The ISAG conference in 2012 outlined the first microsatellite panel of 24 markers for parentage verification: IGF1, S0002, S0005, S0026, S0068, S0090, S0101, S0155, S0178, S0215, S0225, S0226, S0227, S0228, S0355, S0386, SW024, SW072, SW240, SW632, SW857, SW911, SW936, SW951 [18]. An updated list of recommended markers was released in 2014. The STRs were divided into core and additional panels. Fifteen microsatellite loci made up the main panel: S0005, S0090, S0101, S0155, S0227, S0228, S0355, S0386, SW24, SW240, SW72, SW857, SW911, SW936 and SW951. The additional panel includes seven microsatellites: IGF1, S0002, S0026, S0215, S0225, S0226 and SW632 [19]. In the study, we analyzed DNA microsatellite marker polymorphisms of the core STR panel [18,19] in Polish native Puławska pigs and three commercial pig breeds: Polish Large White, Polish Landrace and a foreign breed, Duroc.

The National Breeding Program includes the following breeds: Puławska pig (PUL), Polish Large White (PLW), Polish Landrace (PL) and foreign breeds Duroc (DU), Hampshire and Pietrain. In Poland, these breeds are used for crossbreeding and fattening in pig production and are some of the most economically important (https://www.polsus.pl/index.php/en/pig-breeding, accessed on 29 November 2022). The aims of this study were to assess the level of genetic diversity and determine the population structure of the native Puławska pig and three commercial pig breeds, PL, PLW and DUR, by using a set of 14 STRs. The 14 STRs are recommended for individual pig identification and parentage verification. No studies present the assessment of the polymorphisms of STR markers recommended for the identification of pigs in the Polish population. The study by Szmatola et al. [20] was based only on five STRs not used in routine testing. With the values adjusted for sample sizes, they discovered four breeds to have high levels of genetic diversity: 0.740 for Polish Landrace, 0.697 for Pietrain, 0.692 for Polish Large White and 0.688 for Puławska. However, the Duroc breed has the smallest amount of effective alleles, allelic richness and genetic diversity (0.589). Their findings indicate there has been some gene flow between breeds, particularly between Polish Landrace and Polish Large White. The Duroc breed was shown to have the lowest admixture, confirming its great purity. The authors conclude that further research should probably be performed using more microsatellites and by analyzing mitochondrial DNA. Here, we test the possibility of using 14 STR markers to predict the pig breed, which may be useful in the future for meat traceability.

## 2. Materials and Methods

### 2.1. Material

Blood samples were collected from pigs undergoing routine parentage testing at NRIAP. A total of 338 pigs were studied, including Puławska pigs (PUL, *n* = 85) and three selected commercial breeds: Polish Large White (PLW, *n* = 74), Polish Landrace (PL, *n* = 85) and Duroc (DUR, *n* = 84).

DNA was extracted from blood samples using the Sherlock AX Kit (A&A Biotechnology, Gdynia, Poland), following the manufacturer’s protocol. Extracted DNA was quantified using a NanoDrop 2000 spectrophotometer (Thermo Scientific, Wilmington, DE, USA). 

In the analysis, we selected 14 loci from the recommended ISAG main panel of 15 markers for the identification of individuals and parentage testing in the pig: S0090, S0101, S0155, S0227, S0228, S0355, S0386, SW24, SW240, SW72, SW857, SW911, SW936 and SW951. The markers and used primer sequences are presented by Radko et al. [20,21].

### 2.2. Methods

The one-multiplex reaction containing the 14 STR loci was amplified using the Type-It Microsatellite PCR Kit (Qiagen Inc, Hilden, Germany) reagents and fluorescently labeled primers. The reaction mixtures, with a final reagent volume of 12.5 μL, contained 50 ng DNA. The Veriti^®^ Thermal Cycler amplifier was used for the PCR reaction (Applied Biosystems, Foster City, CA, USA) with the following thermal profile: 5 min initial denaturation at 95 °C, followed by 28 cycles of denaturation at 95 °C for 30 s, annealing at 57 °C for 90 s, elongation of primers at 72 °C for 30 s and final elongation of primers at 60 °C for 30 min. The PCR products were analyzed using an ABI 3500xl capillary sequencer (Applied Bio-Systems, Foster City, CA, USA). The amplified DNA fragments were subjected to electrophoresis in 7% denaturing POP-7 polyacrylamide gel in the presence of a size standard of 500 LIZ (Thermo Fisher Scientific) and a reference sample with a known DNA profile for allele standardization. The results of the electrophoretic separation were analyzed using the GeneMapper^®^ Software 4.0 (Applied Biosystems, Foster City, CA, USA).

### 2.3. Data Analysis

Analysis of molecular variance and genetic differentiation.

Analysis of molecular variance (AMOVA) and pairwise estimates of genetic differentiation (*F*_ST_) across populations were performed using the GenAlEx ver. 6.51 software [21]. 

#### Population Structure and Genetic Distance

Population structure was analyzed using a Bayesian clustering algorithm implemented in STRUCTURE software version 2.3.4 [22,23,24], considering an admixture model with correlated allele frequencies between breeds. The lengths of the burn-in and Monte Carlo Markov Chain (MCMC) simulations were 100,000 and 500,000, respectively, in 5 runs for each number of clusters (K) ranging between 2 and 5. The results were exported to STRUCTURE HARVESTER [25] to plot the likelihood membership coefficient (ΔK) values. 

Genetic distance was analyzed using pairwise estimates of genetic differentiation—FST and Reynolds distance—Ɵw [26]. The individual-animal-based neighbor-joining dendrogram was generated from the estimated pairwise genetic distances between shared alleles using the DARwin ver. 6 software (http://darwin.cirad.fr/, accessed on 29 November 2022).

The population relationships based on principal coordinate analysis (PCoA) were obtained using the GenAlEx ver. 6.51 software [21]. 

## 3. Results and Interpretation

The development of reliable molecular tools for genetically distinguishing between two breeds of species and identifying breed components in food products has become increasingly important due to the increasing demand for improved quality control. For the purpose of the identification of animals and products, microsatellites (STRs) are widely used as molecular markers. STR markers used in this context have to present high diversity. The genetic diversity of microsatellite loci is determined based on genetic parameters such as the PIC index and heterozygosity. These parameter values show the usefulness of markers for further research, including individual identification and genetic population diversity. Previous studies have shown that all 14 STR markers, recommended by ISAG for pig identification, were polymorphic in the sampled groups [27]. Based on the STRs, we calculated the medium genetic differentiation for the breeds studied. Interestingly, the average value of heterozygosity (HE) and the polymorphic information content (PIC) were above 0.5 for all breeds except DUR (PIC = 0.477) [27]. These polymorphism results demonstrate the potential of the analyzed STRs for the individual identification of pigs.

The F-statistic is commonly chosen for studying population structures. It is frequently applied to decompose the genetic variance into within-individual, within-population and among-population components [28]. The analysis of molecular variance (AMOVA) is commonly implemented for estimating the F-statistic [29,30]. 

### 3.1. Analysis of Molecular Variance (AMOVA)

AMOVA is an important element of molecular analysis that allows the statistical inference of genetic variation among and within populations. In our study, the variance analysis (AMOVA) was performed using all 14 polymorphic STRs and revealed that variation among individuals was greater than the variation in the inter-population.

**Figure 1:**
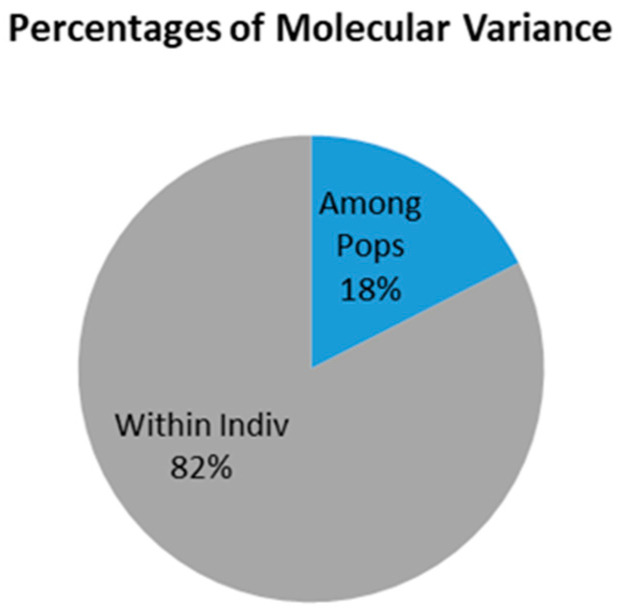
Percentages of AMOVA based on 14 STR markers.

The average genetic differentiation between the breeds was 18% (*p* < 0.001), while the total variability was 82%. Details of AMOVA results are presented in Table 1 and Figure 1. 

In the pig population studied, the AMOVA revealed that most of the variance was attributed to differences within populations, among individuals, and 18% could be attributed to differences among the four pig groups. A similar genetic differentiation was observed with other breeds in other studies using STR markers [13,29].

### 3.2. Structure Analysis

STRUCTURE is the first approach giving an insight into the population structure resulting from the sample set and providing a prelude to other genetic analyses. Assigning individuals to breeds is often useful in population genetics studies, in which obtaining a population classification can provide an inference of individual ancestry that may not have been adequately defined beforehand [22,31,32]. The population structure and degree of admixture of the four pig breeds were evaluated using Bayesian model-based clustering in the STRUCTURE software. The structure of the breeds studied was determined based on the degree of admixture for each individual using the correlated allele frequencies model implemented in the software STRUCTURE. The obtained ΔK value was highest at K = 4. 

**Figure 2:**
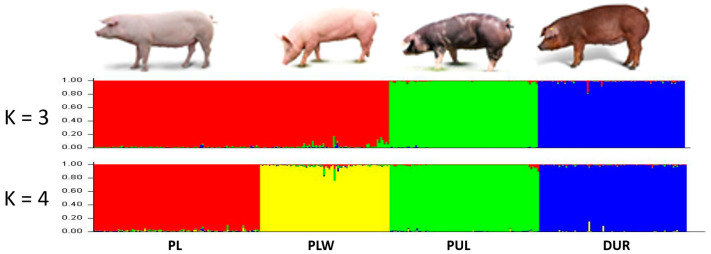
STRUCTURE analysis of 14 STR genotypes from pigs studied. The samples were grouped by the 4 breeds (K = 4). PLW—Polish Large White; PL—Polish Landrace; PUL—Puławska Pig; DUR—Duroc.

**Figure 3:**
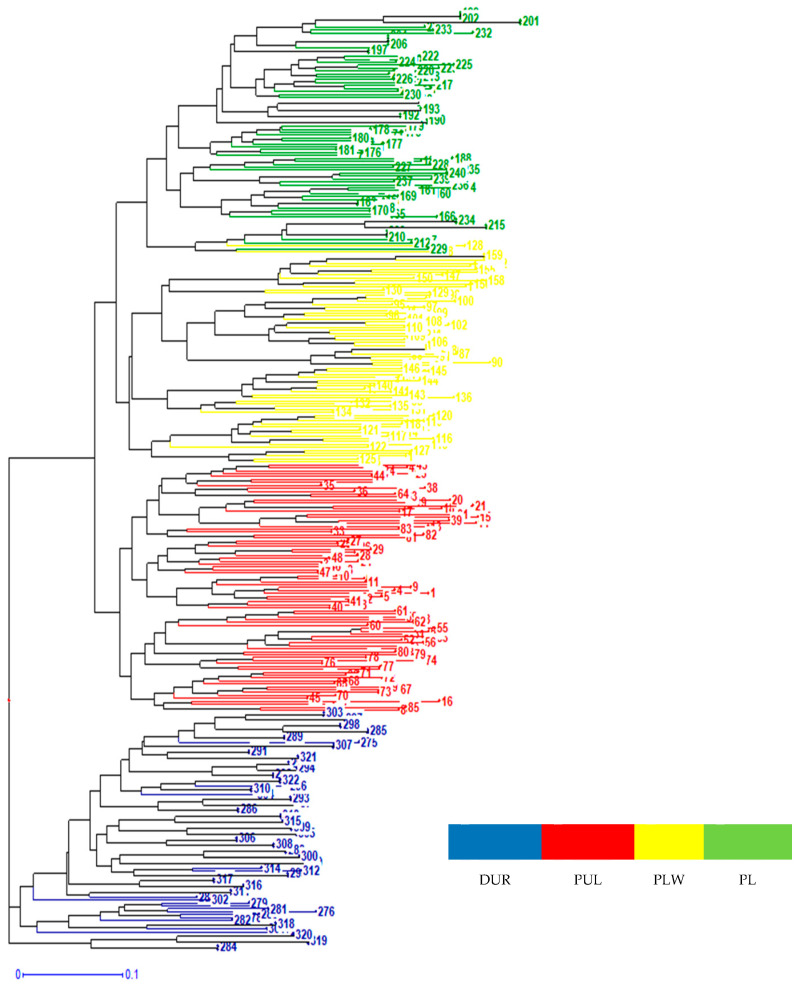
Genetic relationships among pigs with the use of a neighbor-joining tree obtained from a method based on genetic distance. PLW—Polish Large White; PL—Polish Landrace; PUL—Puławska Pig; DUR—Duroc.

On the basis of the 14 STRs, the results of STRUCTURE revealed the subdivision of the pig breeds into four genetic clusters (Figure 2). The average proportion of assignment to the cluster of above 95% was found for all pig breeds. The highest assignment value was found in the DUR (Q = 0.9716). Such a high probability may allow the assignment of unknown individuals to particular breeds. The between-individual tree of Figure 3 shows the same results—four clusters grouping the individuals that belong to the same breed.

### 3.3. Genetic Differentiation

The study of the genetic differentiation of the breeds derived by the population structure analysis considered measures of two common estimates of differentiation—F*_ST_* and the Reynolds genetic distance (Ɵw). The pairwise F*_ST_* values between breeds varied from 0.106 (between PL and PLW) to 0.283 (between PUL and DUR). Similarly, genetic distance was the greatest between PUL and DUR (Ɵw = 0.430) and the smallest between PL and PLW (Ɵw = 0.109) (Table 2). 

The pairwise F*_ST_* and the Reynolds distances among the breeds showed that the national PL and PLW breeds formed one cluster, while Duroc was relatively distant from the other breeds. This indicated that Poland’s pig breeds are separated by the largest genetic distance from the foreign Duroc breed. This reflects the fact that Duroc originated in the USA by the crossing of Red Guinea pigs and Iberian pigs with the Berkshire breed and was introduced to Poland relatively recently—in the 1970s. 

**Figure 4:**
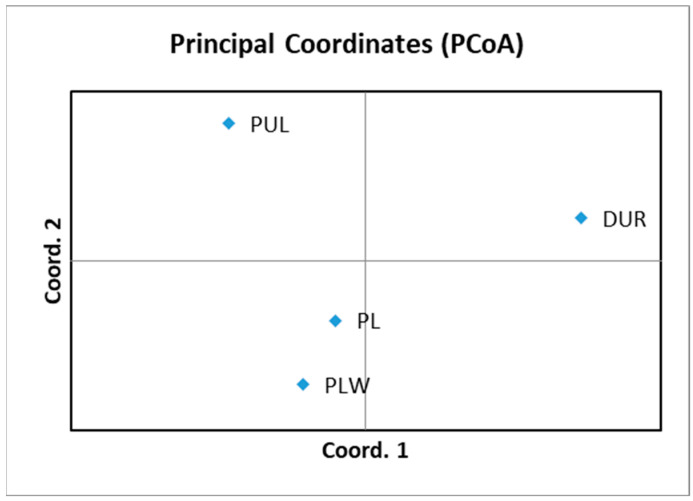
Principal coordinate analysis (PCoA) of the four pig breeds. DUR—Duroc; PUL—Puławska Pig; PL—Polish Landrace; PLW—Polish Large White. A two dimensional plot of the PCoA analysis show the clustering of four breeds. The first and second coordinates account for 56.9% and 24.5%, respectively, of the total variation.

The close genetic relationship between the PL and PLW breeds was proven by STRUCTURE analysis with K = 3 (Figure 2), the pairwise F*_ST_* and Ɵw values. This was further supported by the results of the principal coordinate analysis (PCoA). The obtained results of PCoA analysis show four pig clusters—Duroc (DUR), Puławska (PUL) and the Polish Landrace (PL) and Polish Large White (PLW) together (Figure 4). The PL and PLW breeds were included in one cluster, which confirmed the genetic relationship between these breeds and confirmed the clear distinction of the DUR breed from the Polish population. 

Figure 4 illustrates the population relationships based on the PCoA using 14 STR markers. The first principal coordinate distinguished clearly the DUR breed from the PUL, PL and PLW breeds. The second principal coordinate separated the PL and PLW breeds from the other two pig breeds. The first, second and third principal coordinates (PCoA) represented 56.9%, 24.5% and 18.6%, respectively, of the total variation.

Nowadays, single-nucleotide polymorphism markers are increasingly used for biodiversity studies and the identification and parentage control of livestock, including pigs [33,34,35,36]. SNP genotyping has been used already to develop an SNP panel for discriminating breeds, meat and other pig products [37,38]. However, STR markers are still widely used in routine studies due to their reliability, sensitivity and cheaper analysis methods. Therefore, STR analysis in the pig population should continue.

## 4. Conclusions

The present study analyzed the genetic differentiation of selected pig breeds, Polish Large White, Polish Landrace, Puławska pigs and Duroc, and the possibility of using DNA tests for pig breed prediction.

The analysis of the genetic structure of the pig populations based on 14 STRs showed a clear division of the population into four groups, representing the four selected breeds for study. Our study demonstrates that a panel of microsatellite markers recommended by ISAG for the individual identification of pigs also may be useful for pig breed prediction and, in the future, for meat traceability. It is especially important for the population of native or local pigs, such as the Puławska breed, which can provide meat that is both high in quality and functional.

The presented work can be the first step to developing a system to determine whether meat comes from a pig of the declared breed and whether the meat was produced consistently with the declaration on the packaging. 

## Figures and Tables

**Table 1:** Analysis of molecular variance (AMOVA) based on 14 STR markers. The AMOVA result revealed that individual variation was greater (82%) than the variation in the inter-population (18%).

Source of Variation	df	Sum of Squares	Variance Components	% Variation
Among populations	3	481.869	0.957	18%
Within populations	328	1396.500	4.258	82%
Total	655	3112.256	5.512	100%

AMOVA on model base population of four pig breeds (PLW—Polish Large White; PL—Polish Landrace; PUL—Puławska Pig; DUR—Duroc); df—degree of freedom.

**Table 2:** Reynolds genetic distance (Ɵw) and pairwise estimates of genetic differentiation (*F*_ST_) among 4 studied pig breeds (PUL—Puławska; PLW—Polish Large White; PL—Polish Landrace; DUR—Duroc). The Ɵw values are above the diagonal, and *F*_ST_ estimates are below the diagonal.

	PL	PLW	PUL	DUR
PL		0.109	0.275	0.422
PLW	0.106		0.288	0.429
PUL	0.146	0.147		0.430
DUR	0.201	0.222	0.283	

## Data Availability

The data presented in this study are available within the article.
